# Use of antibiotics in the health care unit: 2015 Pelotas Birth Cohort

**DOI:** 10.11606/s1518-8787.2019053001477

**Published:** 2019-10-10

**Authors:** Andrea Dâmaso Bertoldi, Grégore Iven Mielke, Marília Cruz Guttier, Nelson Arns Neumann, Caroline Dalabona, Alexandra Crispim Boing, Mariângela Freitas Silveira

**Affiliations:** I Universidade Federal de Pelotas. Faculdade de Medicina. Programa de Pós-graduação em Epidemiologia. Pelotas, RS, Brasil; II University of Queensland. School of Human Movement and Nutrition Sciences. Brisbane, Australia; III Coordenação Nacional da Pastoral da Criança. Curitiba, PR, Brazil.; IV Universidade Federal de Santa Catarina. Programa de Pós-graduação em Saúde Coletiva. Florianópolis, SC, Brasil

**Keywords:** Child Care, Anti-Bacterial Agents, administration & dosage, Primary Health Care, Cohort Studies

## Abstract

**OBJECTIVE:**

To estimate the use of the first dose of antibiotics in the health care unit in children from the 2015 Pelotas Birth Cohort at 24 months.

**METHODS:**

A total of 4,014 children were monitored. We used descriptive statistics and Poisson regression to analyze the association between socioeconomic and demographic variables, participation in daycare units, in the activities of the Pastoral da Criança and in the *Primeira Infância Melhor* program, low birth weight, hospitalization between 12 and 24 months, place of medical appointment, prevalence of medical appointment in the last 30 days, prescription of antibiotics, and administration of the first dose in the health care unit.

**RESULTS:**

A total of 1,044 children had medical appointments in the last 30 days, of which 45% were prescribed antibiotics and only 10.5% were administered the first dose of this medication in the health care unit. Children with brown, yellow or indigenous skin color were administered 2.5 times more antibiotics than white children. Children whose mothers had 12 years or more of education were administered 83.0% fewer antibiotics than those whose mothers had up to 4 years of education. Among those who were hospitalized for 12 to 24 months, the use of antibiotics was almost four times higher than among those who were not. Among the children served by the Brazilian Unified Health System (SUS), only 15.3% were administered the first dose of antibiotic in the health care unit. When compared with children served by private health care or health plan, administration of the first dose in the SUS was 76.0% higher.

**CONCLUSIONS:**

Despite the efforts related to the Pastoral da Criança campaign “Antibiotic: first dose immediately,” adherence to the provision of antibiotics in the health care unit is still low. Strategies are necessary and urgent so children have access to the first dose of antibiotics in the health care unit.

## INTRODUCTION

Mortality in childhood is a public health problem, and acute infections constitute one of the main causes of mortality in this age group^1^ . In Brazil, the mortality rate in childhood had an important reduction between 1990 and 2015. Thus, the country fulfilled the goal established in the Millennium Development Goals (MDGs) of reducing by two thirds the mortality of children aged up to five years^2 , 3^ . The rate, which was 53.7 deaths per 1,000 live births in 1990, decreased to 13.8 deaths per 1,000 live births in 2015^3^ .

The main causes of death in childhood include infections, mainly respiratory infections^2 , 4^ . According to data from the Mortality Information System (MIS), respiratory diseases correspond to almost 20.0% of the preventable causes of death in children aged 1–4 years and 6.2% in children aged below 1 year^1^ . Between 1996 and 2016, there was a significant reduction in mortality by respiratory diseases, which decreased 70.0% (data available in Datasus).

Among the sick children taken to health services, most present with respiratory infections without severity, but which may evolve to more severe infections, such as pneumonia and septicemia, if not treated in time^5^ . Therefore, it is essential to distinguish, among children with acute respiratory infection, those with high probability of developing pneumonia. With this better definition of the severity of the disease, the prescription of antibiotics and the need for hospitalization can be defined more appropriately^4^ .

In 2003, the Brazilian Ministry of Health launched the Integrated Management of Childhood Illness strategy (IMCI) with the objective of decreasing the morbidity and mortality of children aged 2 months to 5 years. The strategy was implemented by improving the quality of care provided by health professionals to children, especially in primary care, bringing the recommendations for evaluation and treatment of the main causes of diseases in this age group^5^ This approach was initially developed by the World Health Organization and the United Nations Children’s Fund (UNICEF), with a simultaneous and integrated approach to the set of diseases with the highest prevalence in childhood^1 , 5 , 7^ .

Considering that in Brazil there is a high rate of infant mortality and hospitalization due to bacterial diseases, especially respiratory ones, the *Pastoral da Criança* , a community organization that works in actions to combat infant mortality and improve the quality of life of children, with support from the Ministry of Health, launched in 2011 a permanent campaign called “Antibiotic: first dose immediately.” This campaign aims to warn about the importance of the first dose of antibiotics shortly after the medical appointment, especially in cases of children with suspected pneumonia.

It is estimated that, with effective implementation of the campaign, up to 4,000 deaths of children by bacterial infection would be avoided every year^8^ . According to the Basic Care Supplement number 28, antibiotics should be available in basic health units (BHU), aiming at the administration of the first dose in this same location in cases of pneumonia in children^9^ . A cross-sectional study conducted in 2010 in Rio Grande, RS, evaluated 264 children served in primary health care units and found that only 4.0% of them were administered the first dose of antibiotics in the health care unit, noting that this is not a common practice in the basic health units of that municipality^4^ .

Thus, this study aimed to describe the prevalence of use of the first dose of antibiotics in the health care unit at 24 months in children that participated in the 2015 Pelotas Birth Cohort and to contribute to the campaign follow-up.

## METHODS

The sample of this study includes children belonging to the birth cohort of Pelotas, in the state of Rio Grande do Sul, of 2015, when they were completing 24 months of age. All live births, in the five maternity wards of the municipality, of mothers living in the urban area of Pelotas, between January 1st and December 31, 2015, were eligible to participate in the cohort.

According to the 2010 demographic census, Pelotas is located at the extreme south of Brazil, with an urban population of 328,000 inhabitants, according to the Brazilian Institute of Geography and Statistics (IBGE)^10^ . In order to maintain comparability with previous cohorts in the same municipality, we also included in the sample mothers living in the *Jardim América* neighborhood, currently belonging to another municipality, and in a small fishing village considered as rural zone^11^ .

The children were monitored in the perinatal period and at 3, 12, and 24 months. The follow-up at 24 months was conducted between January and December 2017.

In the perinatal period, we collected information about the child’s sex and the mother’s skin color (reported) and education. The skin color was worked using three categories: white, black and brown/yellow/indigenous (colors grouped into a single category due to the low frequency of individuals corresponding to each of them). In addition, information regarding low birth weight (yes or no) and prematurity (yes or no) was collected in this follow-up. In the follow-up at 24 months, we collected information about maternal age (< 20 years, 20–29 years, 30 years or more), family income (tercile), attendance in daycare unit or school (yes or no), participation in the *Pastoral da Criança* (yes or no), and participation in the *Infância Melhor* program (yes or no).

At 24 months, we collected information about hospitalization of the child aged 12–24 months (yes or no) and place of medical appointment (Unified Health System or private health care/health plan). For this article, we analyzed three dichotomous outcomes: a) medical appointment in the last 30 days due to health problems; b) prescription of antibiotics in medical appointment with physician in the last 30 days; and c) administration of the first dose of antibiotic in the health care unit.

A descriptive analysis of the sample from the cohort was performed at 24 months, in which we estimated the proportion of children who had had a medical appointment in the last 30 days, the proportion of children who needed use of antibiotics among those who had had a medical appointment in the last 30 days and, among these, the proportion of children who had been administered the first dose of antibiotic in the health care unit. Subsequently, bivariate analyses and adjusted analyses were conducted to determine the factors associated with the administration of the first dose of antibiotic in the medical appointment. We used Poisson regression to estimate crude and adjusted prevalence ratios (PR), and conducted the adjusted analysis using the stepwise variable selection model to obtain predictive models and determine the variables that best explain each of the outcomes analyzed. For statistical significance, p < 0.05 was considered. For the prevalence ratios of each predictor presented, we estimated their corresponding 95% confidence intervals (95%CI). The analyses were performed in Stata 12.0.

This study was approved by the Research Ethics Committee of the School of Physical Education of the Federal University of Pelotas (CAAE – protocol 26746414.5.0000.5313), and the informed consent form was signed at the beginning of each follow-up.

## RESULTS

A total of 4,333 women bore children in Pelotas in 2015, and 4,275 (98.7%) agreed to participate in the study. During the follow-up at 24 months, we conducted 4,014 interviews (follow-up rate of 93.9%).

It was found that almost half of the mothers were aged between 20 and 29 years, with white skin color (70.7%), and 65.3% had at least nine years of education. In addition, 9.6% of the children were born with low weight and 7.9% of them were hospitalized in the second year of life. The prevalence of medical appointments in the last 30 days was 26.1%, most (59.2%) in the Unified Health System (SUS). Of those who had medical appointments (n = 1,044), 44.8% received prescription of antibiotics. Among those who were prescribed antibiotics, only 10.5% were administered the first dose in the health care unit ( Table 1 ).

**Table 1:** Description of the sample of children from the 2015 Pelotas Birth Cohort with follow-up at 24 months of age. Pelotas, RS, 2017. (n = 4,014)

Variable	n	%
Child sex		
Male	2,030	50.6
Female	1,984	49.4
Mother’s skin color (reported)		
White	2,832	70.7
Black	637	15.9
Brown, yellow or indigenous	539	13.5
Mother’s age (years)		
< 20	261	6.5
20–29	1,809	45.1
≥ 30	1,942	48.4
Mother’s education (years)		
0–4	356	8.9
5–8	1,036	25.8
9–11	1,386	34.5
12 or more	1,235	30.8
Family income		
Tercile 1	1,342	34.0
Tercile 2	1,370	34.7
Tercile 3	1,236	31.3
Low birth weight		
No	3,628	90.4
Yes	383	9.6
Prematurity		
No	3,423	85.3
Yes	591	14.7
Child attends daycare unit or school		
No	2,940	73.3
Yes	1,071	26.7
Child participates in the Pastoral da Criança		
No	3,891	97.0
Yes	119	3.0
Child participates in the PIM		
No	3,635	90.7
Yes	374	9.3
Child was hospitalized between 12 and 24 months		
No	3,692	92.1
Yes	315	7.9
Child had medical appointment in the last 30 days		
No	2,964	73.9
Yes	1,044	26.1
Place of medical appointment (n = 1,044)		
SUS	617	59.2
Private health care or health plan	426	40.8
Physician prescribed antibiotic (n = 1,044)		
No	575	55.2
Yes	467	44.8
First dose in the health care unit (n = 467)		
No	418	89.5
Yes	49	10.5

PIM: *Primeira Infância Melhor* Program

The prevalence of factors associated with appointment in the last 30 days showed that the frequency of girls was 11% lower than that of boys (p = 0.024). We also found significant association between maternal education (p = 0.005), with 21% higher frequency of appointments for children whose mothers had 12 years or more of education than among children whose mothers had up to 4 years of education (PR = 1.21; 95%CI 0.99–1.48). Children who attended daycare unit or school and who were hospitalized between 12 and 24 months of age were more likely to having had an appointment in the 30 days prior to the interview (PR = 1.5; 95%CI 1.35–1,66 and PR = 1.37; 95%CI 1.16–1.61, respectively) ( Table 2 ).

**Table 2:** Prevalence and prevalence ratios (PR) of medical appointments in the last 30 days according to independent variables. Children from the 2015 Pelotas Birth Cohort with follow-up at 24 months. Pelotas, RS, 2017. (n = 4,014)

Variable	n (%)	Crude analysis	

		PR (95%CI)	p
Child sex			0.024
Male	560 (27.6)	1.00	
Female	484 (24.5)	0.89 (0.80–0.98)	
Mother’s skin color (reported)			0.838
White	741 (26.2)	1.00	
Black	160 (25.1)	0.96 (0.83–1.11)	
Brown, yellow or indigenous	142 (26.4)	1.01 (0.86–1.17)	
Mother’s age (years)			0.053
< 20	56 (21.5)	1.00	
20–29	453 (25.1)	1.17 (0.91–1.49)	
≥ 30	535 (27.5)	1.28 (1.01–1.64)	
Mother’s education (years)			0.005
0–4	87 (24.4)	1.00	
5–8	241 (23.3)	0.95 (0.77–1.18)	
9–11	352 (25.4)	1.04 (0.85–1.28)	
12 or more	364 (29.6)	1.21 (0.99–1.48)	
Family income			0.226
Tercile 1	331 (24.7)	1.00	
Tercile 2	355 (25.9)	1.05 (0.92–1.19)	
Tercile 3	342 (27.7)	1.12 (0.98–1.28)	
Low birth weight			0.098
No	958 (26.5)	1.00	
Yes	86 (22.5)	0.85 (0.70–1.03)	
Prematurity			0.382
No	899 (26.3)	1.00	
Yes	145 (24.6)	0.93 (0.80–1.09)	
Child attends daycare unit or school			< 0.001
No	676 (23.0)	1.00	
Yes	368 (34.4)	1.50 (1.35–1.66)	
Child participates in the Pastoral da Criança			0.221
No	1,018 (26.2)	1.00	
Yes	25 (21.0)	0.80 (0.56–1.14)	
Child participates in the PIM			0.505
No	952 (26.2)	1.00	
Yes	92 (24.6)	0.94 (0.78–1.13)	
Child was hospitalized between 12 and 24 months			< 0.001
No	934 (25.3)	1.00	
Yes	109 (34.6)	1.37 (1.16–1.61)	

PIM: *Primeira Infância Melhor* Program

Children of mothers with educational level between 9 and 11 years of education showed the lowest prevalence of antibiotic prescription (40.5%). Low birth weight and participation in the *Primeira Infância Melhor* (PIM) program showed a positive association with the prescription of this medication. Low birth weight children had 36.0% more prescriptions than normal birth weight children (PR = 1.36; 95%CI 1.13–1.65), as well as those who participated in the PIM (PR = 1.24; 95%CI 1.01–1,51) ( Table 3 ).

**Table 3:** Prevalence and prevalence ratios (PR) of antibiotic prescription in medical appointment in the last 30 days according to independent variables. Children from the 2015 Pelotas Birth Cohort with follow-up at 24 months. Pelotas, RS, 2017. (n = 1,044)

Variable	n (%)	Crude analysis	

		PR (95%CI)	p
Child sex			0.217
Male	260 (46.6)	1.00	
Female	207 (42.8)	0.51 (0.41–0.62)	
Mother’s skin color (reported)			0.779
White	332 (44.9)	1.00	
Black	68 (42.5)	0.95 (0.78–1.15)	
Brown, yellow or indigenous	66 (46.5)	1.03 (0.85–1.26)	
Mother’s age (years)			0.928
< 20	24 (42.9)	1.00	
20–30	205 (45.4)	1.06 (0.77–1.46)	
≥ 30	238 (44.6)	1.04 (0.76–1.43)	
Mother’s education (years)			0.037
0–4	49 (56.3)	1.00	
5–8	112 (46.5)	0.83 (0.66–1.04)	
9–11	142 (40.5)	0.72 (0.57–0.90)	
12 or more	164 (45.2)	0.80 (0.65–1.00)	
Family income			0.554
Tercile 1	148 (44.9)	1.00	
Tercile 2	152 (42.8)	0.95 (0.81–1.13)	
Tercile 3	160 (46.9)	1.05 (0.89–1.23)	
Low birth weight			0.001
No	416 (43.5)	1.00	
Yes	51 (59.3)	1.36 (1.13–1.65)	
Prematurity			0.264
No	396 (44.2)	1.00	
Yes	71 (49.0)	1.11 (0.92–1.33)	
Child attends daycare unit or school			0.112
No	290 (43.0)	1.00	
Yes	177 (48.1)	1.12 (0.97–1.28)	
Child participates in the Pastoral da Criança			0.938
No	455 (44.8)	1.00	
Yes	11 (44.0)	0.98 (0.63–1.54)	
Child participates in the PIM			0.037
No	417 (43.9)	1.00	
Yes	50 (54.5)	1.24 (1.01–1.51)	
Child was hospitalized between 12 and 24 months			0.194
No	412 (44.2)	1.00	
Yes	55 (50.5)	1.14 (0.93–1.39)	
Place of medical appointment (n = 1,044)			0.865
SUS	275 (44.6)	1.00	
Private health care or health plan	192 (45.2)	1.01 (0.88–1.16)	

PIM: *Primeira Infância Melhor* Program

As for the prevalence of antibiotic use in the health care unit, it was observed that people with brown, yellow or indigenous skin color had 2.5 times higher use than those with white skin color (PR = 2.52; 95%CI 1.40–4.52). Children whose mothers had 12 years or more of education were administered 83.0% fewer antibiotics than children whose mothers had up to 4 years of education (PR = 0.17; 95%CI 0.05–0.56; p = 0.002). A negative association can be observed between family income and antibiotic use in the health care unit, 87.0% lower among children of mothers in the highest tercile of income (p < 0.001). In addition, children who attended daycare unit or school also showed lower antibiotic use in the health care unit (PR = 0.47; 95%CI 0.25–0.90), while those hospitalized between 12 and 24 months showed almost four times higher antibiotic use than those who were not hospitalized (PR = 3.98; 95%CI 2.37–6.67). Among the children served in the SUS who received antibiotic prescription, only 15.3% were administered the first dose of antibiotic in the medical appointment. This prevalence was 76.0% higher than among children served in private health care or health plan units (PR = 0.24; 95%CI 0.11–0.52) ( Table 4 ).

**Table 4:** Prevalence and prevalence ratios (PR) of use of the first dose of antibiotic in the health care unit according to independent variables. Children from the 2015 Pelotas Birth Cohort with follow-up at 24 months. Pelotas, RS, 2017. (n = 467)

Variable	n (%)	Crude analysis	

		PR (95%CI)	p
Child sex			0.602
Male	29 (11.2)	1.00	
Female	20 (9.7)	0.87 (0.50–1.49)	
Mother’s skin color (reported)			0.008
White	28 (8.4)	1.00	
Black	7 (10.3)	1.22 (0.56–2.68)	
Brown, yellow or indigenous	14 (21.2)	2.52 (1.40–4.52)	
Mother’s age (years)			0.158
< 20	5 (20.8)	1.00	
20–30	23 (11.2)	0.54 (0.23–1.29)	
≥ 30	21 (8.8)	0.42 (0.18–1.02)	
Mother’s education (years)			0.002
0–4	7 (14.3)	1.00	
5–8	21 (18.8)	1.31 (0.60–2.89)	
9–11	17 (12.0)	0.84 (0.37–1.90)	
12 or more	4 (2.4)	0.17 (0.05–0.56)	
Family income			< 0.001
Tercile 1	28 (18.9)	1.00	
Tercile 2	16 (10.5)	0.56 (0.31–0.99)	
Tercile 3	4 (2.5)	0.13 (0.05–0.37)	
Low birth weight			0.193
No	41 (9.9)	1.00	
Yes	8 (15.7)	1.59 (0.79–3.21)	
Prematurity			0.514
No	10 (10.1)	1.00	
Yes	9 (12.7)	1.25 (0.64–2.47)	
Child attends daycare unit or school			0.023
No	38 (13.1)	1.00	
Yes	11 (6.2)	0.47 (0.25–0.90)	
Child participates in the Pastoral da Criança			0.388
No	47 (10.3)	1.00	
Yes	2 (18.2)	1.76 (0.49–6.36)	
Child participates in the PIM			0.387
No	42 (10.1)	1.00	
Yes	7 (14.0)	1.39 (0.66–2.93)	
Child was hospitalized between 12 and 24 months			< 0.001
No	32 (7.8)	1.00	
Yes	17 (30.9)	3.98 (2.37–6.67)	

PIM: *Primeira Infância Melhor* Program

Table 5 presents the variables that best explain the variability of outcomes after adjusted analyses. As for medical appointment in the 30 days prior to the interview, the frequency of girls was 10% lower than that of boys (PR = 0.90; 95%CI 0.81–1.00), that of children who attended daycare unit or school was about 1.5 times higher than that of children who did not attend (PR = 1.48; 95%CI 1.33–1.64) and that of children who were hospitalized was 42.0% higher than among those who were not hospitalized (PR = 1.42; 95%CI 1.21–1.68). The variables presented explain 19% of the variability of the outcome ( Table 5 ).

**Table 5:** Adjusted analysis with predictive model including the variables that most explain the outcomes.

Variable	PR (95%CI)	p	Adjusted R^2^
Medical appointment in the last 30 days (n = 4,002)			19.0%
Child sex		0.045	
Male	1.00		
Female	0.90 (0.81–1.00)		
Mother’s age (years)		0.081	
< 20	1.00		
20–30	1.13 (0.88–1.45)		
≥ 30	1.21 (0.94–1.55)		
Low birth weight		0.113	
No	1.00		
Yes	0.86 (0.71–1.04)		
Child attends daycare unit or school		< 0.001	
No	1.00		
Yes	1.48 (1.33–1.64)		
Child was hospitalized between 12 and 24 months		< 0.001	
No	1.00		
Yes	1.42 (1.21–1.68)		
Prescription of antibiotic (n = 1,026)			12.7%
Child sex		0.199	
Male	1.00		
Female	0.91 (0.80–1.05)		
Mother’s education (years)		0.042	
0–4	1.00		
5–8	0.85 (0.67–1.08)		
9–11	0.73 (0.57– 0.93)		
12 or more	0.76 (0.58– 0.99)		
Family income		0.103	
Tercile 1	1.00		
Tercile 2	1.02 (0.86–1.22)		
Tercile 3	1.18 (0.97–1.45)		
Low birth weight		0.003	
No	1.00		
Yes	1.35 (1.11–1.64)		
Child attends daycare unit or school		0.050	
No	1.00		
Yes	1.16 (1.00–1.35)		
Child participates in the PIM		0.022	
No	1.00		
Yes	1.27 (1.04–1.57)		
First dose in the health care unit (n = 459)			11.0%
Mother’s skin color (reported)		0.035	
White	1.00		
Black	0.99 (0.47–2.11)		
Brown, yellow or indigenous	2.08 (1.12–3.88)		
Mother’s education (years)		0.089	
0–4	1.00		
5–8	1.35 (0.62–2.92)		
9–11	0.99 (0.44–2.25)		
12 or more	0.37 (0.11–1.24)		
Family income		0.021	
Tercile 1	1.00		
Tercile 2	0.76 (0.42–1.37)		
Tercile 3	0.27 (0.09–0.84)		
Child was hospitalized between 12 and 24 months		< 0.001	
No	1.00		
Yes	3.59 (2.11–6.10)		

PIM: *Primeira Infância Melhor* Program

As for antibiotic prescription, the child’s sex, maternal education, family income, low birth weight, frequency in daycare unit or school, and participation in the PIM were able to explain 12.7% of the variability of the outcome. After adjustment, they remained significantly associated with maternal education (p = 0.042), low birth weight (p = 0.003) and participation in the PIM (p = 0.022) ( Table 5 ).

The model that best predicts the use of the first dose of antibiotic in the health care unit includes maternal skin color, maternal education, family income, and hospitalization between 12 and 24 months of age, explaining 11.0% of the variability of the outcome ( Table 5 ). For children of women with brown, yellow, or indigenous skin color, administration of the first dose in the health care unit was about two times more frequent than among mothers with white skin color (PR = 2.08; 95%CI 1.12– 3.88). Children in the highest tercile of income showed the lowest antibiotic use in the health care unit (PR = 0.27; 95%CI 0.09–0.84). Finally, children who were hospitalized between 12 and 24 months received 3.6 times more medication in the health care unit than those who were not hospitalized (PR = 3.59; 95%CI 2.11–6.10) ( Table 5 ).

## DISCUSSION

This study found that almost half of the children who had had medical appointments in the last 30 days received prescription for antibiotic, and only 10.5% of them were administered the first dose in the health care unit. In addition, when comparing the children served in the SUS and those served by private health care or health plan, the frequency among the first ones was 76% higher.

An important part of the children had medical appointments in the last 30 days, with most being served by the SUS, as can be observed in practically the entire national territory^12^ . Health services, particularly in primary care, have an important role in the Brazilian context, especially because children aged below 5 years present as the main causes of hospitalizations the sensitive problems for this level of care^15^ . The National Policy on Primary Care (NPPC) and the National Policy for Integral Child Health Care (NPICHC) are efforts to improve child health and have contributed to this. NPPC includes children’s health in the care lines, conducting effective interventions with the purpose of improving it^16 , 17^ .

In addition to health services, maternal characteristics are also related to children’s health. Maternal education constitutes an impactful factor in health outcomes^15^ , and low levels of maternal education can be considered a risk factor for children’s health^18^ . This study found that higher level of maternal education was positively associated with a greater number of medical appointments in the last 30 days, which may be considered a protective factor for children’s health, by the resort to care and access to the health service.

It was found that the children attending daycare units had 48% more medical appointments when compared with those who did not attend them. This fact can be explained by the greater exposure of the children to pathogens in these closed environments starting from the six months of age, which facilitates the onset of infections, especially respiratory infections, for which the use of antibiotics is necessary^19 , 20^ . A study conducted by Bricks et al.^12^ in São Paulo found that 80% of the children aged below 2 years who attended daycare units had already been administered medications, with antibiotics being the most frequent. Children aged below 2 years presented a relative risk of 2.9 (95%CI 2.6–3.3) for the use of medications when compared with children of other ages. In addition, the authors found that about 25% of the children aged below 2 years were administered five or more medications. In the other age groups, the administration of five or more medications did not reach 5%^12^ . The study of Oliveira et al.^21^ , using data from this cohort, demonstrated an association between the frequency in daycare units and the higher occurrence of symptoms and infectious morbidities at the 12 months of life of the child.

Among the prescribed medications, antibiotics are part of a class widely used in children aged up to five years^19 , 20^ , which indicates the need to promote their rational use, which ensures the therapeutic success and contributes to decrease the development of bacterial resistance^19^ . However, for that to occur, it is necessary that the medicine is correctly selected, at the appropriate dose for the age group, and for the appropriate period of time, always seeking to initiate treatment as soon as possible to avoid the worsening of the health status of the patient.

Researches conducted in different parts of the world^22 , 23^ indicate a high prevalence of antibiotic use among children. In Brazil, a study conducted in São Paulo also found that almost half of the antibiotic prescriptions were for children aged below 5 years^19^ . In Bagé, RS, in 2007, 36% of the children aged up to 1 year and 42% of the children aged 2–5 years had prescription of antibiotics^24^ , results that corroborate the findings of this study.

A study conducted in Jataí, GO, found that the prevalence of antibiotic use in the last month in children aged 0–4 years was 11%, reaching 59% and for those in the income range of 1–3 minimum wages^25^ . In the present study, using educational level as socioeconomic indicator, it was found that children whose mothers had higher educational level had lower prevalence of antibiotic prescription.

In addition, low birth weight and PIM were associated with higher prevalence of antibiotic prescription. This can be explained by the profile of children born with low birth weight, who have a higher risk of negative health outcomes, and of PIM participants, who are also children in a more vulnerable situation^26^ .

Despite the high prevalence of antibiotic use, it is observed low prevalence of administration of the first dose in the health care unit. A study conducted in southern Brazil observed that only 4% of the children were administered the first dose of the drug in the BHU immediately after the medical appointment^4^ . Furthermore, the researchers found that only 4% of the physicians said they always adopted this conduct, suggesting that this practice is not yet followed by most professionals^4^ .

Although administration of the first dose of antibiotics in the SUS is 76% higher compared with children served by private health care or health plan, its prevalence of 15% can be considered very low, since there is an incentive program to that end. Some hypotheses may be propounded for this situation, such as the non-availability of the medication in the BHU, since more than 50% of the individuals with prescription originating from the SUS are not provided all medications^27^ , and problems in the flow of care service and in the adherence to clinical protocols.

Access to the first dose of antibiotic in the health care unit was associated with skin color, maternal education, family income and hospitalization in the last year, showing that access to this drug in the heath care unit is affecting the most vulnerable population, which rely mostly on the SUS^27^ .

The management of acute respiratory infection has been a priority in recent decades, with the implementation of control and prevention programs^5 , 7 , 28^ . Training in Integrated Management of Childhood Illness (IMCI) to classify diseases that really require antibiotics can be critical for better care. A study conducted in Brazil observed significant difference between professionals with and without training in IMCI in the assessment of the child, classification of the disease, treatment and communication with parents or caregivers^30 , 31^ .

Pharmacists can also be a great allies. Studies have shown that the presence of this professional in the emergency service may increase the probability of the patient receiving the appropriate dose of antibiotic and in a more timely manner^32 ,33^, demonstrating their aid in the rational use of antibiotics. However, in Brazil, this professional is still underutilized in health services.

The limitations of the study were not considering the reason for the use of antibiotics in order to evaluate whether the indications were correct, not classifying the place of the medical appointment as hospital or outpatient clinic, and not determining whether the professionals who served the children had had the IMCI training. However, it can be observed that 60% of the prescriptions were antibiotics indicated for respiratory diseases. As for the place of medical appointment, almost all the children received the medication at the outpatient level, since only 17 of those who received the first dose of antibiotic in the health care unit in the last 30 days had been hospitalized in the last 12 months. As for the non-evaluation of the professionals who served these children as to IMCI training, it is presumed that, regardless of training, because the campaign was implemented in 2011, all professionals and health units in 2018 should be prepared to administer the first dose in the health care unit.

## CONCLUSION

This study showed that, despite the efforts of the Ministry of Health in conjunction with the *Pastoral da Criança* in the campaign “Antibiotic: first dose immediately,” adherence to its provision in the health care unit is still low. Although the SUS presents better performance than the private health care or health plan, the prevalence is still very low. The findings suggest that actions to stimulate the children’s access to the first dose of antibiotics are essential, as they avoid the worsening of the disease, hospitalization, and even death. The service organization and health professionals should also ensure access to the first dose in the health care unit to avoid the worsening of the condition.

## References

[1] 1. Ministério da Saúde (BR), Secretaria de Atenção à Saúde, Departamento de Atenção Básica. Recomendação sobre a administração da primeira dose de antibiótico para crianças com diagnóstico de pneumonia, nas unidades de saúde. Brasília, DF; 2010.

[2] 2. França EB, Lansky S, Rego MAS, Malta DC, França JS, Teixeira R, et al. Leading causes of child mortality in Brazil, in 1990 and 2015: estimates from the Global Burden of Disease study. Rev Bras Epidemiol. 2017;20 Supl1:46-60. 10.1590/1980-5497201700050005 28658372

[3] 3. Secretaria de Comunicação (BR), Portal Brasil. O Brasil e os ODM. Brasília, DF; 2018 [cited 2019 Jan 20]. Available from: http://www.odmbrasil.gov.br/o-brasil-e-os-odm

[4] 4. Costa LR, Silva LCM, Cesar JA. Administração da primeira dose do medicamento nos serviços de saúde: um estudo com menores de cinco anos de idade no extremo-sul do Brasil. Rev AMRIGS. 2013;57(2):117-21.

[5] 5. Ministério da Saúde (BR); Organização Pan-americana de Saúde; Fundo das Nações Unidas para a Infância. Manual AIDPI criança: 2 meses a 5 anos. Brasília, DF: Ministério da Saúde; 2017.

[6] 6. Corrêa RA, São José BP, Malta DC, Passos VMA, França EB, Teixeira RA, et al. Carga de doença por infecções do trato respiratório inferior no Brasil, 1990 a 2015: estimativas do estudo Global Burden of Disease 2015. Rev Bras Epidemiol. 2017;20 Supl 1:171-81. 10.1590/1980-5497201700050014 28658381

[7] 7. Figueiras AC, Souza ICN, Rios VG, Benguigui Y. Manual para vigilância do desenvolvimento infantil no contexto da AIDPI. Washington, DC: Organização Pan-Americana da Saúde; 2005.

[8] 8. Wieczorkievicz AAM, Soares P, Junkes CHG. Realidade e desafios das ESFs para a execução da primeira dose imediata de antibiótico para crianças em situação de doença. Saude Meio Ambiente. 2016;5(1):78-88.

[9] 9. Ministério da Saúde (BR), Secretaria de Atenção à Saúde, Departamento de Atenção Básica. Acolhimento demanda espontânea. Brasília - DF: Ministério da Saúde; 2013. (Cadernos de Atenção Básica, 28, v.1).

[10] 10. Instituto Brasileiro de Geografia e Estatística. Cidades: Pelotas, RS. Rio de Janeiro: IBGE; 2018 [cited 2019 Jan 20]. Available from: https://cidades.ibge.gov.br/brasil/rs/pelotas/panorama

[11] 11. Hallal PC, Bertoldi AD, Domingues MR, Silveira MF, Demarco FF, Silva ICM, et al. Cohort Profile: The 2015 Pelotas (Brazil) Birth Cohort Study. Int J Epidemiol. 2018;47(4):1048. 10.1093/ije/dyx219 PMC6124621 29126133

[12] 12. Bricks LF, Leone C. Utilização de medicamentos por crianças atendidas em creches. Rev Saude Publica. 1996;30(6):527-35. 10.1590/S0034-89101996000600006 9302822

[13] 13. Stopa SR, Malta DC, Monteiro CN, Szwarcwald CL, Goldbaum M, Cesar CLG. Use of and access to health services in Brazil, 2013 National Health Survey. Rev Saude Publica. 2017;51 Supl 1:3s. 10.1590/s1518-8787.2017051000074 PMC5676359 28591351

[14] 14. Moreira JPL, Moraes JR, Luiz RR. Utilização de consulta médica e hipertensão arterial sistêmica nas áreas urbanas e rurais do Brasil, segundo dados da PNAD 2008. Cienc Saude Coletiva. 2011;16(9):3781-93. 10.1590/S1413-81232011001000014 21987321

[15] 15. Barreto JOM, Nery IS, Costa MSC. Estratégia Saúde da Família e internações hospitalares em menores de 5 anos no Piauí, Brasil. Cad Saude Publica. 2012;28(3):515-26. 10.1590/S0102-311X2012000300012 22415184

[16] 16. Ministério da Saúde (BR), Secretaria de Atenção à Saúde, Departamento de Atenção Básica. Política Nacional de Atenção Básica. Brasília, DF; 2012. (Série E. Legislação em Saúde).

[17] 17. Ministério da Saúde (BR). Portaria nº 2.436, de 21 de setembro de 2017. Aprova a Política Nacional de Atenção Básica, estabelecendo a revisão de diretrizes para a organização da Atenção Básica, no âmbito do Sistema Único de Saúde (SUS). Diario Oficial da União. 22 ago 2017; Seção 1:68.

[18] 18. Haidar FH, Oliveira UF, Nascimento LFC. Escolaridade materna: correlação com os indicadores obstétricos. Cad Saude Publica. 2001;17(4):1025-9. 10.1590/S0102-311X2001000400037 11514884

[19] 19. Carvalho DC, Trevisol FS, Menegali BT, Trevisol DJ. Uso de medicamentos em crianças de zero a seis anos matriculadas em creches de Tubarão, Santa Catarina. Rev Paul Pediatr. 2008;26(3):238-44. 10.1590/S0103-05822008000300007

[20] 20. Oliveira PD, Bertoldi AD, Silva BGC, Domingues MR, Neumann NA, Silveira MF. Day care attendance during the first 12 months of life and occurrence of infectious morbidities and symptoms. J Pediatr. 2018. In press. 10.1016/j.jped.2018.05.012 31679611

[21] 21. Rossignoli A, Clavenna A, Bonati M. Antibiotic prescription and prevalence rate in the outpatient paediatric population: analysis of surveys published during 2000-2005. Eur J Clin Pharmacol. 2007;63(12):1099-106. 10.1007/s00228-007-0376-3 17891535

[22] 22. Gagliotti C, Morsillo F, Resi D, Milandri M, Moro ML. Antibiotic treatments for children ages 0-23 months in a northern Italy region: a cohort study. Infection. 2006;34(3):155-7. 10.1007/s15010-006-5106-8 16804659

[23] 23. Menezes APS, Domingues MR, Baisch ALM. Compreensão das prescrições pediátricas de antimicrobianos em Unidades de Saúde em um município do sul do Brasil. Rev Bras Epidemiol. 2009;12(3):478-89. 10.1590/S1415-790X2009000300016

[24] 24. Braolos A, Pereira ACS, Bizerra AA, Policarpo OF, Soares NC, Barbosa AS. Uso de antimicrobianos pela população da cidade de Jataí (GO), Brasil. Cienc Saude Coletiva. 2013;18(10):3055-60. 10.1590/S1413-81232013001000030 24061032

[25] 25. Martins ALO, Nascimento DSF, Schneider IJC, Schuelter-Trevisol F. Incidence of community-acquired infections of lower airways among infants. Rev Paul Pediatr. 2016;34(2):204-9. 10.1016/j.rppede.2015.10.005 PMC4917272 26987781

[26] 26. Boing AC, Bertoldi AD, Boing AF, Bastos JL, Peres KG. Acesso a medicamentos no setor público: análise de usuários do Sistema Único de Saúde no Brasil. Cad Saude Publica. 2013;29(4):691-701. 10.1590/S0102-311X2013000400007 23568299

[27] 27. Higuchi CH, Fujimori E, Cursino EG, Chiesa AM, Veríssimo MDLÓR, Mello DF. Atenção Integrada às Doenças Prevalentes na Infância (AIDPI) na prática de enfermeiros egressos da USP. Rev Gaucha Enferm. 2011;32(2):241-7. 10.1590/S1983-14472011000200005 21987983

[28] 28. World Health Organization; The United Nations Children’s Fund. Ending preventable child deaths from pneumonia and diarrhoea by 2025: the integrated Global Action Plan for Pneumonia and Diarrohea (GAPPD). Geneva: WHO; UNICEF; 2013.

[29] 29. Benguigui Y. Acute respiratory infections control in the context of the IMCI strategy in the Americas. Rev Bras Saude Mater Infant. 2003;3(1):25-36. 10.1590/S1519-38292003000100005

[30] 30. Amaral J, Gouws E, Bryce J, Leite AJM, Cunha ALA, Victora CG. Effect of Integrated Management of Childhood Illness (IMCI) on health worker performance in Northeast-Brazil. Cad Saude Publica. 2004;20 Supl 2:S209-19. 10.1590/S0102-311X2004000800016 15608935

[31] 31. Bailey AM, Stephan M, Weant KA, Justice SB. Dosing of appropriate antibiotics and time to administration of first doses in the pediatric emergency department. J Pediatr Pharmacol Ther. 2015;20(4):309-15. 10.5863/1551-6776-20.4.309 PMC4557721 26380571

[32] 32. DeFrates SR, Weant KA, Seamon JP, Shirakbari A, Baker SN. Emergency pharmacist impact on health care-associated pneumonia empiric therapy. J Pharm Pract. 2013;26(2):125-30. 10.1177/0897190012451933 22918891

