# Supplementary material for: Use of antibiotics in the health care unit: 2015 Pelotas Birth Cohort
Source: Rev Saude Publica. 2019 Oct 10;53:92. doi: 10.11606/s1518-8787.2019053001477 (PMC9586444; doi:10.11606/s1518-8787.2019053001477)
Supplement: Translation

# Translation: Uso de antibióticos no local do atendimento: Coorte de Nascimentos de Pelotas de 2015

**Authors:** Andrea Dâmaso Bertoldi, Grégore Iven Mielke, Marília Cruz Guttier, Nelson Arns Neumann, Caroline Dalabona, Alexandra Crispim Boing, Mariângela Freitas Silveira

**Affiliations:** I Universidade Federal de Pelotas. Faculdade de Medicina. Programa de Pós-graduação em Epidemiologia. Pelotas, RS, Brasil; II University of Queensland. School of Human Movement and Nutrition Sciences. Brisbane, Australia; III Coordenação Nacional da Pastoral da Criança. Curitiba, PR, Brasil; IV Universidade Federal de Santa Catarina. Programa de Pós-graduação em Saúde Coletiva. Florianópolis, SC, Brasil

**Keywords:** Cuidado da Criança, Antibacterianos, administração & dosagem, Atenção Primária à Saúde, Estudos de Coortes

## Abstract

**OBJETIVO:**

Estimar o uso da primeira dose do antibiótico no local de atendimento nas crianças da Coorte de Nascimentos de Pelotas de 2015 aos 24 meses.

**MÉTODOS:**

Foram acompanhadas 4.014 crianças. A associação entre variáveis socioeconômicas e demográficas, participação em creche, nas ações da Pastoral da Criança e no programa Primeira Infância Melhor, baixo peso ao nascer, internação entre 12 e 24 meses, local da consulta, prevalência de consulta nos últimos 30 dias, prescrição de antibióticos e recebimento da primeira dose no local de atendimento foi analisada por meio de estatística descritiva e regressão de Poisson.

**RESULTADOS:**

Tiveram consulta nos últimos 30 dias 1.044 crianças, das quais 45% receberam prescrição de antibiótico e apenas 10,5% receberam a primeira dose dessa medicação no local de atendimento. Crianças de cor da pele parda, amarela ou indígena tiveram um uso de antibiótico 2,5 vezes maior que o das brancas. Já as crianças cujas mães tinham 12 anos ou mais de escolaridade usaram 83,0% menos antibióticos que aquelas cujas mães tinham até quatro anos de estudo. Entre aquelas que foram internadas entre 12 e 24 meses, o uso de antibiótico foi quase quatro vezes maior do que entre as que não foram. Entre as crianças atendidas pelo Sistema Único de Saúde (SUS), apenas 15,3% receberam a primeira dose do antibiótico no local de atendimento. Quando comparado com o de crianças atendidas por financiamento particular ou convênio, o recebimento da primeira dose no SUS chegou a ser 76,0% superior.

**CONCLUSÕES:**

Apesar dos esforços relacionados à campanha da Pastoral da Criança “Antibiótico: primeira dose imediata”, ainda é baixa a adesão ao fornecimento de antibióticos no local de atendimento. Estratégias são necessárias e urgentes para que as crianças tenham acesso à primeira dose de antibióticos no local de atendimento.

## INTRODUÇÃO

A mortalidade na infância é um problema de saúde pública, sendo as infecções agudas uma das principais causas de mortalidade nessa faixa etária^1^ . No Brasil, a taxa de mortalidade na infância sofreu importante redução entre 1990 e 2015. Com isso, o país cumpriu a meta estabelecida nos Objetivos de Desenvolvimento do Milênio (ODM) de reduzir em dois terços a mortalidade de crianças até cinco anos^2 , 3^ . O índice, que era de 53,7 mortes por mil nascidos vivos em 1990, passou para 13,8 mortes para mil nascidos vivos no ano de 2015^3^ .

Entre as principais causas de óbito na infância estão as infecções, principalmente as respiratórias^2 , 4^ . De acordo com os dados do Sistema de Informações sobre Mortalidade (SIM), as doenças respiratórias correspondem a quase 20,0% das causas de morte evitáveis em crianças de um a quatro anos e 6,2% das crianças menores de um ano^1^ . Entre 1996 e 2016, houve uma redução importante da mortalidade por doenças respiratórias, que chegou a diminuir 70,0% (dados disponíveis no Datasus).

Entre as crianças doentes que buscam os serviços de saúde, a grande maioria apresenta infecções respiratórias sem gravidade, mas que podem evoluir para infecções mais graves, como pneumonia e septicemia, se não forem tratadas a tempo^5^ . Portanto, é essencial distinguir, entre as crianças com infecção respiratória aguda, aquelas com alta probabilidade de desenvolver pneumonia. Com essa melhor definição da gravidade da doença, é possível definir mais adequadamente a prescrição de antibióticos e a necessidade de hospitalização^4^ .

Em 2003, o Ministério da Saúde lançou a estratégia Atenção Integrada às Doenças Prevalentes na Infância (AIDPI Criança) com o objetivo de diminuir a morbidade e a mortalidade de crianças entre dois meses e cinco anos de idade. A estratégia foi instrumentalizada pela melhoria da qualidade da atenção prestada por profissionais de saúde à criança, em especial na atenção básica, trazendo as recomendações para avaliação e tratamento das principais causas de doenças nessa faixa etária^5^ . Essa abordagem foi desenvolvida inicialmente pela Organização Mundial da Saúde e pelo Fundo das Nações Unidas para a Infância (Unicef), com uma abordagem simultânea e integrada do conjunto de doenças de maior prevalência na infância^1 , 5 , 7^ .

Considerando que no Brasil há um grande índice de internação hospitalar e mortalidade infantil por doenças bacterianas, principalmente as respiratórias, a Pastoral da Criança, uma organização comunitária que trabalha em ações para combater a mortalidade infantil e melhorar a qualidade de vida das crianças, com apoio do Ministério da Saúde, lançou em 2011 uma campanha permanente chamada “Antibiótico: primeira dose imediata”. Essa campanha visa alertar sobre a importância da primeira dose dos antibióticos logo após a consulta, principalmente nos casos de crianças com suspeita de pneumonia.

Estima-se que, com a efetivação da campanha, seriam evitadas até quatro mil mortes de crianças por infecção bacteriana todos os anos^8^ . De acordo com os Cadernos de Atenção Básica número 28, antibióticos devem estar disponíveis nas unidades básicas de saúde (UBS), visando a administração da primeira dose nesse mesmo local nos casos de pneumonia em crianças^9^ . Estudo transversal conduzido em 2010, em Rio Grande, RS, avaliou 264 crianças atendidas em unidades básicas de saúde e identificou que apenas 4,0% delas receberam a primeira dose de antibiótico no local de atendimento, constatando que essa não é uma prática comum nas unidades básicas de saúde daquele município^4^ .

Dessa forma, o objetivo do presente estudo foi descrever a prevalência de uso da primeira dose do antibiótico no local de atendimento aos 24 meses nas crianças participantes da Coorte de Nascimentos de Pelotas de 2015 e contribuir com o monitoramento da campanha.

## MÉTODOS

A amostra do presente estudo inclui as crianças pertencentes à Coorte de Nascimentos de Pelotas, RS, de 2015, quando elas estavam completando 24 meses de idade. Foram elegíveis para participar da coorte todos os nascidos vivos, nas cinco maternidades do município, de mães residentes na zona urbana de Pelotas, entre 1º de janeiro e 31 de dezembro de 2015.

De acordo com o censo demográfico de 2010, Pelotas está localizada no extremo sul do Brasil, com população urbana de 328 mil habitantes, segundo o Instituto Brasileiro de Geografia e Estatística (IBGE)^10^ . Para manter a comparabilidade com as coortes anteriores realizadas no mesmo município, foram incluídas na amostra também mães residentes no Bairro Jardim América, atualmente pertencente a outro município, e numa pequena vila de pescadores considerada como zona rural^11^ .

As crianças foram acompanhadas no período perinatal e aos 3, 12 e 24 meses. O acompanhamento dos 24 meses foi realizado entre janeiro e dezembro de 2017.

No período perinatal foram coletadas informações sobre sexo da criança, cor da pele da mãe (relatada) e escolaridade materna. A cor da pele foi trabalhada utilizando três categorias: branca, preta e parda/amarela/indígena (cores agrupadas em uma única categoria devido à baixa frequência de indivíduos correspondentes a cada uma). Além disso, neste acompanhamento foram coletadas informações relativas ao baixo peso ao nascer (sim ou não) e prematuridade (sim ou não). No acompanhamento aos 24 meses foram coletadas as informações sobre idade materna (< 20 anos, 20–29 anos, 30 anos ou mais), renda familiar (tercil), frequência em creche ou escolinha (sim ou não), participação na Pastoral da Criança (sim ou não) e participação no Programa Infância Melhor (sim ou não).

Aos 24 meses foram coletadas informações sobre internação da criança entre 12 e 24 meses de idade (sim ou não) e local de consulta (Sistema Único de Saúde ou particular/convênio). Para o presente artigo, três desfechos dicotômicos foram analisados: a) consulta médica nos últimos 30 dias devido a problemas de saúde; b) prescrição de antibiótico na consulta com médico nos últimos 30 dias; e c) recebimento da primeira dose do antibiótico no local de atendimento.

Foi realizada uma análise descritiva da amostra da coorte aos 24 meses, na qual se calculou a proporção de crianças que tiveram consulta nos últimos 30 dias, proporção de crianças que necessitavam de uso de antibióticos entre as que tiveram consulta nos últimos 30 dias e, entre essas, proporção das que receberam a primeira dose do antibiótico no local de atendimento. Posteriormente, foram conduzidas análises bivariadas e análises ajustadas para identificar os fatores associados ao recebimento da primeira dose de antibiótico no local da consulta. Para o cálculo de razão de prevalência (RP) bruta e ajustada, utilizou-se a regressão de Poisson, enquanto a análise ajustada foi conduzida utilizando modelo de seleção de variáveis *stepwise* , com o objetivo de obter modelos preditivos e identificar as variáveis que melhor explicam cada um dos desfechos analisados. Para a significância estatística foi considerado p < 0,05. Para as razões de prevalência de cada preditor apresentado, foram calculados seus respectivos intervalos de confiança de 95% (IC95%). As análises foram realizadas no software Stata 12.0.

Este estudo foi aprovado pelo Comitê de Ética em Pesquisa da Faculdade de Educação Física da Universidade Federal de Pelotas (CAAE – Protocolo 26746414.5.0000.5313), e o termo de consentimento livre esclarecido foi assinado no início de cada acompanhamento.

## RESULTADOS

No total, 4.333 mulheres tiveram filhos em Pelotas no ano de 2015, e 4.275 (98,7%) aceitaram participar do estudo. Durante o acompanhamento de 24 meses foram conduzidas 4.014 entrevistas (taxa de acompanhamento de 93,9%).

Verificou-se que quase metade das mães tinha idade entre 20 e 29 anos, com cor de pele branca (70,7%) e 65,3% tinham pelo menos nove anos de estudo. Além disso, 9,6% das crianças nasceram com baixo peso e 7,9% delas foram internadas no segundo ano de vida. A prevalência de consultas médicas nos últimos 30 dias foi de 26,1%, a maioria (59,2%) realizada no Sistema Único de Saúde (SUS). Das que tiveram consultas médicas (n = 1.044), 44,8% receberam prescrição de antibióticos. Entres as que receberam prescrição de antibióticos, apenas 10,5% receberam a primeira dose no local de atendimento ( Tabela 1 ).

**Tabela 1:** Descrição da amostra de crianças da Coorte de Nascimentos de Pelotas de 2015 acompanhadas aos 24 meses de idade. Pelotas, RS, 2017. (n = 4.014)

Variável	n	%
Sexo da criança		
Masculino	2.030	50,6
Feminino	1.984	49,4
Cor da pele da mãe (relatada)		
Branca	2.832	70,7
Preta	637	15,9
Parda, amarela ou indígena	539	13,5
Idade da mãe (anos)		
< 20	261	6,5
20–29	1.809	45,1
≥ 30	1.942	48,4
Escolaridade da mãe (anos)		
0–4	356	8,9
5–8	1.036	25,8
9–11	1.386	34,5
12 ou mais	1.235	30,8
Renda familiar		
Tercil 1	1.342	34,0
Tercil 2	1.370	34,7
Tercil 3	1.236	31,3
Baixo peso ao nascer		
Não	3.628	90,4
Sim	383	9,6
Prematuridade		
Não	3.423	85,3
Sim	591	14,7
Criança frequenta creche ou escolinha		
Não	2.940	73,3
Sim	1.071	26,7
Criança participa da Pastoral da Criança		
Não	3.891	97,0
Sim	119	3,0
Criança participa do PIM		
Não	3.635	90,7
Sim	374	9,3
Criança foi internada entre os 12 e os 24 meses		
Não	3.692	92,1
Sim	315	7,9
Criança teve consulta médica nos últimos 30 dias		
Não	2.964	73,9
Sim	1.044	26,1
Local de consulta (n = 1.044)		
SUS	617	59,2
Particular ou convênio	426	40,8
Médico receitou antibiótico (n = 1.044)		
Não	575	55,2
Sim	467	44,8
Primeira dose no local de atendimento (n = 467)		
Não	418	89,5
Sim	49	10,5

PIM: Programa Primeira Infância Melhor

Quando analisada a prevalência de fatores associados com a consulta nos últimos 30 dias, observou-se que a frequência das meninas foi 11% menor que a dos meninos (p = 0,024). Também foi detectada associação significativa entre escolaridade materna (p = 0,005), com uma frequência de consultas em crianças cujas mães tinham 12 anos ou mais de estudo 21% mais do que entre as crianças cujas mães tinham até 4 anos de escolaridade (RP = 1,21; IC95% 0,99–1,48). Crianças que frequentavam creche ou escolinha e que foram internadas entre 12 e 24 meses de idade tiveram maior probabilidade de consulta nos 30 dias anteriores à entrevista (RP = 1,5; IC95% 1,35–1,66 e RP = 1,37; IC95% 1,16–1,61, respectivamente) ( Tabela 2 ).

**Tabela 2:** Prevalência e razões de prevalência (RP) de consulta nos últimos 30 dias conforme variáveis independentes. Crianças da Coorte de Nascimentos de Pelotas de 2015 acompanhadas aos 24 meses. Pelotas, RS, 2017. (n = 4.014)

Variável	n (%)	Análise bruta	

		RP (IC95%)	p
Sexo da criança			0,024
Masculino	560 (27,6)	1,00	
Feminino	484 (24,5)	0,89 (0,80–0,98)	
Cor da pele da mãe (relatada)			0,838
Branca	741 (26,2)	1,00	
Preta	160 (25,1)	0,96 (0,83–1,11)	
Parda, amarela ou indígena	142 (26,4)	1,01 (0,86–1,17)	
Idade da mãe (anos)			0,053
< 20	56 (21,5)	1,00	
20–29	453 (25,1)	1,17 (0,91–1,49)	
≥ 30	535 (27,5)	1,28 (1,01–1,64)	
Escolaridade da mãe (anos)			0,005
0–4	87 (24,4)	1,00	
5–8	241 (23,3)	0,95 (0,77–1,18)	
9–11	352 (25,4)	1,04 (0,85–1,28)	
12 ou mais	364 (29,6)	1,21 (0,99–1,48)	
Renda familiar			0,226
Tercil 1	331 (24,7)	1,00	
Tercil 2	355 (25,9)	1,05 (0,92–1,19)	
Tercil 3	342 (27,7)	1,12 (0,98–1,28)	
Baixo peso ao nascer			0,098
Não	958 (26,5)	1,00	
Sim	86 (22,5)	0,85 (0,70–1,03)	
Prematuridade			0,382
Não	899 (26,3)	1,00	
Sim	145 (24,6)	0,93 (0,80–1,09)	
Criança frequenta creche ou escolinha			< 0,001
Não	676 (23,0)	1,00	
Sim	368 (34,4)	1,50 (1,35–1,66)	
Criança participa da Pastoral da Criança			0,221
Não	1.018 (26,2)	1,00	
Sim	25 (21,0)	0,80 (0,56–1,14)	
Criança participa do PIM			0,505
Não	952 (26,2)	1,00	
Sim	92 (24,6)	0,94 (0,78–1,13)	
Criança foi internada entre os 12 e os 24 meses			< 0,001
Não	934 (25,3)	1,00	
Sim	109 (34,6)	1,37 (1,16–1,61)	

PIM: Programa Primeira Infância Melhor

Filhos de mães com escolaridade entre 9 e 11 anos de estudo foram as que tiveram menor prevalência de prescrição de antibióticos (40,5%). Baixo peso ao nascer e participação no programa Primeira Infância Melhor (PIM) apresentaram associação positiva com a prescrição desse medicamento. As crianças com baixo peso ao nascer tiveram 36,0% mais prescrições do que aquelas que nasceram com peso normal (RP = 1,36; IC95% 1,13–1,65), bem como aquelas que participaram do PIM (RP = 1,24; IC95% 1,01–1,51) ( Tabela 3 ).

**Tabela 3:** Prevalência e razões de prevalência (RP) de prescrição de antibiótico na consulta com médico nos últimos 30 dias conforme variáveis independentes. Crianças da Coorte de Nascimentos de Pelotas de 2015 acompanhadas aos 24 meses. Pelotas, RS, 2017. (n = 1.044)

Variável	n (%)	Análise bruta	

		RP (IC95%)	p
Sexo da criança			0,217
Masculino	260 (46,6)	1,00	
Feminino	207 (42,8)	0,51 (0,41–0,62)	
Cor da pele da mãe (relatada)			0,779
Branca	332 (44,9)	1,00	
Preta	68 (42,5)	0,95 (0,78–1,15)	
Parda, amarela ou indígena	66 (46,5)	1,03 (0,85–1,26)	
Idade da mãe (anos)			0,928
< 20	24 (42,9)	1,00	
20–30	205 (45,4)	1,06 (0,77–1,46)	
≥ 30	238 (44,6)	1,04 (0,76–1,43)	
Escolaridade da mãe (anos)			0,037
0–4	49 (56,3)	1,00	
5–8	112 (46,5)	0,83 (0,66–1,04)	
9–11	142 (40,5)	0,72 (0,57–0,90)	
12 ou mais	164 (45,2)	0,80 (0,65–1,00)	
Renda familiar			0,554
Tercil 1	148 (44,9)	1,00	
Tercil 2	152 (42,8)	0,95 (0,81–1,13)	
Tercil 3	160 (46,9)	1,05 (0,89–1,23)	
Baixo peso ao nascer			0,001
Não	416 (43,5)	1,00	
Sim	51 (59,3)	1,36 (1,13–1,65)	
Prematuridade			0,264
Não	396 (44,2)	1,00	
Sim	71 (49,0)	1,11 (0,92–1,33)	
Criança frequenta creche ou escolinha			0,112
Não	290 (43,0)	1,00	
Sim	177 (48,1)	1,12 (0,97–1,28)	
Criança participa da Pastoral da Criança			0,938
Não	455 (44,8)	1,00	
Sim	11 (44,0)	0,98 (0,63–1,54)	
Criança participa do PIM			0,037
Não	417 (43,9)	1,00	
Sim	50 (54,5)	1,24 (1,01–1,51)	
Criança foi internada entre os 12 e os 24 meses			0,194
Não	412 (44,2)	1,00	
Sim	55 (50,5)	1,14 (0,93–1,39)	
Local de consulta (n = 1.044)			0,865
SUS	275 (44,6)	1,00	
Particular ou convênio	192 (45,2)	1,01 (0,88–1,16)	

PIM: Programa Primeira Infância Melhor

Em relação à prevalência de uso de antibióticos no local de atendimento, observou-se que as pessoas de cor da pele parda, amarela ou indígena tiveram um uso 2,5 vezes maior que as de pele branca (RP = 2,52; IC95% 1,40–4,52). As crianças cujas mães tinham 12 anos ou mais de escolaridade usaram 83,0% menos que aquelas crianças cujas mães tinham até 4 anos de estudo (RP = 0,17; IC95% 0,05–0,56; p = 0,002). É possível observar associação negativa entre a renda familiar e utilização do antibiótico no local de atendimento, 87,0% menor entre as crianças de mães no maior tercil de renda (p < 0,001). Além disso, crianças que frequentavam creche ou escolinha também utilizaram menos antibiótico no local de atendimento (RP = 0,47; IC95% 0,25–0,90), enquanto as que foram internadas entre 12 e 24 meses utilizaram quase quatro vezes mais do que as que não foram internadas (RP = 3,98; IC95% 2,37–6,67). Entre as crianças atendidas pelo SUS que receberam prescrição de antibiótico, apenas 15,3% receberam a primeira dose do antibiótico no local da consulta. Essa prevalência foi 76,0% maior do que entre as crianças cujo local de atendimento tinha financiamento particular ou por convênio (RP = 0,24; IC95% 0,11–0,52) ( Tabela 4 ).

**Tabela 4:** Prevalência e razões de prevalência (RP) de uso da primeira dose do antibiótico no local de atendimento conforme variáveis independentes. Crianças da Coorte de Nascimentos de Pelotas de 2015 acompanhadas aos 24 meses. Pelotas, RS, 2017. (n = 467)

Variável	n (%)	Análise bruta	

		RP (IC95%)	p
Sexo da criança			0,602
Masculino	29 (11,2)	1,00	
Feminino	20 (9,7)	0,87 (0,50–1,49)	
Cor da pele da mãe (relatada)			0,008
Branca	28 (8,4)	1,00	
Preta	7 (10,3)	1,22 (0,56–2,68)	
Parda, amarela ou indígena	14 (21,2)	2,52 (1,40–4,52)	
Idade da mãe (anos)			0,158
< 20	5 (20,8)	1,00	
20–30	23 (11,2)	0,54 (0,23–1,29)	
≥ 30	21 (8,8)	0,42 (0,18–1,02)	
Escolaridade da mãe (anos)			0,002
0–4	7 (14,3)	1,00	
5–8	21 (18,8)	1,31 (0,60–2,89)	
9–11	17 (12,0)	0,84 (0,37–1,90)	
12 ou mais	4 (2,4)	0,17 (0,05–0,56)	
Renda familiar			< 0,001
Tercil 1	28 (18,9)	1,00	
Tercil 2	16 (10,5)	0,56 (0,31–0,99)	
Tercil 3	4 (2,5)	0,13 (0,05–0,37)	
Baixo peso ao nascer			0,193
Não	41 (9,9)	1,00	
Sim	8 (15,7)	1,59 (0,79–3,21)	
Prematuridade			0,514
Não	10 (10,1)	1,00	
Sim	9 (12,7)	1,25 (0,64–2,47)	
Criança frequenta creche ou escolinha			0,023
Não	38 (13,1)	1,00	
Sim	11 (6,2)	0,47 (0,25–0,90)	
Criança participa da Pastoral da Criança			0,388
Não	47 (10,3)	1,00	
Sim	2 (18,2)	1,76 (0,49–6,36)	
Criança participa do PIM			0,387
Não	42 (10,1)	1,00	
Sim	7 (14,0)	1,39 (0,66–2,93)	
Criança foi internada entre os 12 e os 24 meses			< 0,001
Não	32 (7,8)	1,00	
Sim	17 (30,9)	3,98 (2,37–6,67)	

PIM: Programa Primeira Infância Melhor

Na Tabela 5 estão apresentadas as variáveis que melhor explicam a variabilidade dos desfechos após análises ajustadas. Com relação à consulta nos 30 dias anteriores à entrevista, a frequência de meninas foi 10% menor que a de meninos (RP = 0,90; IC95% 0,81–1,00), a de crianças que frequentavam creche ou escolinha foi cerca de 1,5 vezes maior que a de crianças que não frequentavam (RP = 1,48; IC95% 1,33–1,64) e a de crianças que foram internadas foi 42,0% maior do que entre aquelas que não internaram (RP = 1,42; IC95% 1,21–1,68). As variáveis apresentadas explicam 19% da variabilidade do desfecho ( Tabela 5 ).

**Tabela 5:** Análise ajustada com modelo preditivo incluindo as variáveis que mais explicam os desfechos.

Variável	RP (IC95%)	p	R^2^ajustado
Consulta nos últimos 30 dias (n = 4.002)			19,0%
Sexo da criança		0,045	
Masculino	1,00		
Feminino	0,90 (0,81–1,00)		
Idade da mãe (anos)		0,081	
< 20	1,00		
20–30	1,13 (0,88–1,45)		
≥ 30	1,21 (0,94–1,55)		
Baixo peso ao nascer		0,113	
Não	1,00		
Sim	0,86 (0,71–1,04)		
Criança frequenta creche ou escolinha		< 0,001	
Não	1,00		
Sim	1,48 (1,33–1,64)		
Criança foi internada entre os 12 e os 24 meses		< 0,001	
Não	1,00		
Sim	1,42 (1,21–1,68)		
Prescrição de antibiótico (n = 1.026)			12,7%
Sexo da criança		0,199	
Masculino	1,00		
Feminino	0,91 (0,80–1,05)		
Escolaridade da mãe (anos)		0,042	
0–4	1,00		
5–8	0,85 (0,67–1,08)		
9–11	0,73 (0,57– 0,93)		
12 ou mais	0,76 (0,58– 0,99)		
Renda familiar		0,103	
Tercil 1	1,00		
Tercil 2	1,02 (0,86–1,22)		
Tercil 3	1,18 (0,97–1,45)		
Baixo peso ao nascer		0,003	
Não	1,00		
Sim	1,35 (1,11–1,64)		
Criança frequenta creche ou escolinha		0,050	
Não	1,00		
Sim	1,16 (1,00–1,35)		
Criança participa do PIM		0,022	
Não	1,00		
im	1,27 (1,04–1,57)		
Primeira dose no local (n = 459)			11,0%
Cor da pele da mãe (relatada)		0,035	
Branca	1,00		
Preta	0,99 (0,47–2,11)		
Parda, amarela ou indígena	2,08 (1,12–3,88)		
Escolaridade da mãe (anos)		0,089	
0–4	1,00		
5–8	1,35 (0,62–2,92)		
9–11	0,99 (0,44–2,25)		
12 ou mais	0,37 (0,11–1,24)		
Renda familiar		0,021	
Tercil 1	1,00		
Tercil 2	0,76 (0,42–1,37)		
Tercil 3	0,27 (0,09–0,84)		
Criança foi internada entre os 12 e os 24 meses		< 0,001	
Não	1,00		
Sim	3,59 (2,11–6,10)		

PIM: Programa Primeira Infância Melhor

Com relação à prescrição de antibiótico, o sexo da criança, escolaridade materna, renda da família, baixo peso ao nascer, frequência em creche ou escolinha e participação no PIM foram capazes de explicar 12,7% da variabilidade do desfecho. Após ajuste, mantiveram-se significativamente associados a escolaridade materna (p = 0,042), baixo peso ao nascer (p = 0,003) e participação no PIM (p = 0,022) ( Tabela 5 ).

O modelo que melhor prediz o uso da primeira dose de antibiótico no local de atendimento inclui cor da pele materna, escolaridade materna, renda familiar e internação entre 12 e 24 meses de idade, explicando 11,0% da variabilidade do desfecho ( Tabela 5 ). Para filhos de mulheres de cor de pele parda, amarela ou indígena, receber a primeira dose no local de atendimento foi cerca de duas vezes mais frequente do que entre os de mães brancas (RP = 2,08; IC95% 1,12–3,88). Crianças no maior tercil de renda foram as que menos utilizaram antibiótico no local de atendimento (RP = 0,27; IC95% 0,09–0,84). Finalmente, crianças que forma internadas entre 12 e 24 meses receberam 3,6 vezes mais o medicamento no local de atendimento do que as que não foram (RP = 3,59; IC95% 2,11–6,10) ( Tabela 5 ).

## DISCUSSÃO

O presente estudo identificou que quase a metade das crianças que tiveram consulta médica nos últimos 30 dias receberam prescrição de antibiótico, e apenas 10,5% delas receberam a primeira dose no local de atendimento. Além disso, comparando as crianças atendidas no SUS e as atendidas por financiamento particular ou convênio, a frequência entre as primeiras chegou a ser 76% superior.

Uma importante parcela das crianças passou por consulta médica nos últimos 30 dias, sendo a maior parte atendida pelo SUS, conforme pode ser observado em praticamente todo o território nacional^12^ . Os serviços de saúde, em especial na atenção primária, têm papel importante no contexto brasileiro, especialmente porque as crianças menores de cinco anos apresentam como principais causas de internações hospitalares os problemas sensíveis a esse nível de atenção^15^ . A Política Nacional de Atenção Básica (PNAB) e a Política Nacional de Atenção Integral à Saúde da Criança (PNAISC) são esforços para a melhoria da saúde infantil e vêm contribuindo nesse sentido. A PNAB inclui a saúde da criança nas linhas de cuidado, desenvolvendo intervenções efetivas com o propósito de melhorá-la^16 , 17^ .

Além dos serviços de saúde, as características maternas também têm relação com a saúde das crianças. A escolaridade materna constitui fator impactante nos desfechos em saúde^15^ , com baixos níveis podendo ser considerados um fator de risco para a saúde infantil^18^ . O presente estudo identificou que a escolaridade materna mais elevada se associou positivamente ao maior número de consultas nos últimos 30 dias, o que pode ser considerado um fator de proteção para a saúde da criança, pela busca do atendimento e acesso ao serviço de saúde.

Identificou-se que as crianças que frequentaram creche tiveram 48% mais consultas quando comparadas às que não frequentaram. Este fato pode ser explicado pela maior exposição da criança a patógenos nesses ambientes fechados a partir dos seis meses, o que facilita o aparecimento de infecções, principalmente as respiratórias, para as quais o uso de antibióticos se faz necessário^19 , 20^ . Estudo conduzido por Bricks et al.^12^ em São Paulo identificou que 80% das crianças menores de dois anos que frequentavam creches já haviam utilizado medicamentos, sendo os antibióticos os mais frequentes. As crianças menores de dois anos apresentaram risco relativo de 2,9 (IC95% 2,6–3,3) para a utilização de medicamentos quando comparadas com crianças de outras idades. Além disso, os autores identificaram que cerca de um quarto das crianças com menos de dois anos utilizou cinco ou mais remédios. Já nas outras faixas etárias, a utilização de cinco ou mais medicamentos não chegou a 5%^12^ . O estudo de Oliveira et al.^21^ , utilizando dados desta coorte, demonstrou associação entre a frequência em creches e a maior ocorrência de morbidades infecciosas e sintomas aos 12 meses de vida da criança.

Dentre os medicamentos prescritos, os antibióticos fazem parte de uma classe muito utilizada por crianças de até cinco anos de idade^19 , 20^ , o que indica a necessidade da promoção do seu uso racional, o que garante o sucesso terapêutico e contribui para a diminuição do desenvolvimento da resistência bacteriana^19^ . No entanto, para que isso ocorra, é necessário que o medicamento seja selecionado corretamente, na dose adequada à faixa etária e pelo período de tempo apropriado, buscando sempre iniciar o tratamento o mais breve possível para evitar o agravamento do estado de saúde do paciente.

Pesquisas conduzidas em diferentes partes do mundo^22 , 23^ indicam alta prevalência do uso de antibióticos entre as crianças. No Brasil, estudo realizado em São Paulo identificou também que quase metade das prescrições de antibióticos foram feitas para crianças com menos de cinco anos^19^ . Em Bagé, RS, no ano de 2007, 36% das crianças de até um ano e 42% das crianças entre dois a cinco anos tiveram prescrição de antibióticos^24^ , resultados que corroboram os achados do presente estudo.

Estudo realizado em Jataí, GO, identificou que a prevalência de uso de antibióticos no último mês em crianças de zero a quatro anos foi de 11%, chegando a 59% e para aquelas na faixa de renda de um a três salários mínimos^25^ . No presente estudo, utilizando a escolaridade como indicador socioeconômico, verificou-se que as crianças com mães de maior escolaridade tiveram menor prevalência de prescrição de antibióticos.

Além disso, o baixo peso ao nascer e o PIM mostraram-se associados à maior prevalência de prescrição de antibióticos. Isso pode ser explicado pelo perfil das crianças nascidas com baixo peso, que têm maior risco de desfechos negativos em saúde, e dos participantes do PIM, que são também crianças em situação mais vulnerável^26^ .

Apesar da alta prevalência do uso de antibióticos, observa-se a baixa prevalência da administração da primeira dose no local de atendimento. Estudo conduzido no sul do Brasil observou que apenas 4% das crianças receberam a primeira dose do medicamento na UBS imediatamente após a consulta^4^ . Ademais, os pesquisadores identificaram que apenas 4% dos médicos disseram adotar essa conduta sempre, sugerindo que essa prática ainda não é seguida pela maioria dos profissionais^4^ .

Apesar de a administração da primeira dose de antibióticos no SUS ser 76% superior à das crianças atendidas por financiamento particular ou convênio, sua prevalência de 15% pode ser considerada muito baixa, uma vez que existe um programa de incentivo para isso. Algumas hipóteses podem ser lançadas para essa situação, como a não disponibilização do medicamento na UBS, já que mais de 50% dos indivíduos com prescrição originária do SUS não conseguem receber todos os medicamentos^27^ , e problemas no fluxo de atendimento e na adesão a protocolos clínicos.

O acesso à primeira dose do antibiótico no local de atendimento esteve associado à cor da pele, escolaridade materna, renda familiar e internação hospitalar no último ano, mostrando que o acesso a esse medicamento no local está atingindo a população mais vulnerável, que depende majoritariamente do SUS^27^ .

O manejo da infecção respiratória aguda tem sido prioridade nas últimas décadas, com a implementação de programas de controle e prevenção^5 , 7 , 28^ . O treinamento em AIDPI para classificar as doenças que realmente necessitam de antibióticos pode ser fundamental para o melhor cuidado. Estudo realizado no Brasil observou diferença significativa entre profissionais com e sem treinamento em AIDPI na avaliação da criança, classificação da doença, tratamento e comunicação com os pais ou cuidadores^30 , 31^ .

O profissional farmacêutico pode ser também um grande aliado. Estudos demonstraram que a presença desse profissional no serviço de emergência pode aumentar a probabilidade de o paciente receber a dose adequada do antibiótico e de forma mais oportuna^32 ,33^, demonstrando seu auxílio no uso racional de antibióticos. Entretanto, no Brasil esse profissional ainda é subutilizado nos serviços de saúde.

As limitações do estudo foram não considerar o motivo do uso do antibiótico para poder avaliar se as indicações estavam corretas, não classificar o local de atendimento entre hospitalar e ambulatorial e não constatar se os profissionais que atenderam as crianças haviam realizado o treinamento AIDPI. No entanto, é possível observar que 60% das prescrições eram de antibióticos indicados para doenças respiratórias. Já em relação ao local de atendimento, quase a totalidade das crianças recebeu o medicamento em nível ambulatorial, já que apenas 17 das que receberam a primeira dose de antibiótico no local de atendimento nos últimos 30 dias haviam sido internadas nos últimos 12 meses. Em relação à não avaliação dos profissionais que atenderam essas crianças quanto ao treinamento AIDPI, presume-se que, independentemente do treinamento, como a campanha foi implementada em 2011, todos os profissionais e unidades de saúde em 2018 deveriam estar preparadas para administrar a primeira dose no local de atendimento.

## CONCLUSÃO

O presente estudo mostrou que, apesar dos esforços do Ministério da Saúde conjuntamente com a Pastoral da Criança na campanha “Antibiótico: primeira dose imediata”, ainda é baixa a adesão ao seu fornecimento no local de atendimento. Apesar de o SUS apresentar um desempenho melhor que o atendimento particular ou de convênio, ainda é muito baixa a prevalência. Os achados sugerem que ações para estimular o acesso das crianças à primeira dose de antibiótico é fundamental, pois evitam a piora da doença, uma internação hospitalar e até mesmo a morte. A organização dos serviços e os profissionais da saúde também devem garantir o acesso à primeira dose no local de atendimento para evitar a piora do quadro.
